# Phase I Evaluation of STA-1474, a Prodrug of the Novel HSP90 Inhibitor Ganetespib, in Dogs with Spontaneous Cancer

**DOI:** 10.1371/journal.pone.0027018

**Published:** 2011-11-03

**Authors:** Cheryl A. London, Misty D. Bear, Jennifer McCleese, Kevin P. Foley, Reji Paalangara, Takayo Inoue, Weiwen Ying, James Barsoum

**Affiliations:** 1 Department of Veterinary Biosciences, The Ohio State University, Columbus, Ohio, United States of America; 2 Translational Biology, GlaxoSmithKline, Collegeville, Pennsylvania, United States of America; 3 Intervet/Schering-Plough Animal Health, Summit, New Jersey, United States of America; 4 Drug Metabolism and Pharmacokinetics, Synta Pharmaceuticals Corp., Lexington, Massachusetts, United States of America; 5 Discovery Chemistry, Synta Pharmaceuticals Corp., Lexington, Massachusetts, United States of America; 6 Theracrine, Inc., Cambridge, Massachusetts, United States of America; Roswell Park Cancer Institute, United States of America

## Abstract

**Background:**

The novel water soluble compound STA-1474 is metabolized to ganetespib (formerly STA-9090), a potent HSP90 inhibitor previously shown to kill canine tumor cell lines *in vitro* and inhibit tumor growth in the setting of murine xenografts. The purpose of the following study was to extend these observations and investigate the safety and efficacy of STA-1474 in dogs with spontaneous tumors.

**Methods and Findings:**

This was a Phase 1 trial in which dogs with spontaneous tumors received STA-1474 under one of three different dosing schemes. Pharmacokinetics, toxicities, biomarker changes, and tumor responses were assessed. Twenty-five dogs with a variety of cancers were enrolled. Toxicities were primarily gastrointestinal in nature consisting of diarrhea, vomiting, inappetence and lethargy. Upregulation of HSP70 protein expression was noted in both tumor specimens and PBMCs within 7 hours following drug administration. Measurable objective responses were observed in dogs with malignant mast cell disease (n = 3), osteosarcoma (n = 1), melanoma (n = 1) and thyroid carcinoma (n = 1), for a response rate of 24% (6/25). Stable disease (>10 weeks) was seen in 3 dogs, for a resultant overall biological activity of 36% (9/25).

**Conclusions:**

This study provides evidence that STA-1474 exhibits biologic activity in a relevant large animal model of cancer. Given the similarities of canine and human cancers with respect to tumor biology and HSP90 activation, it is likely that STA-1474 and ganetespib will demonstrate comparable anti-cancer activity in human patients.

## Introduction

Heat shock protein 90 (HSP90), a molecular chaperone that promotes the conformational maturation and stabilization of a wide variety of client proteins, is a promising target for therapeutic intervention in cancer[1], [2], [3], [4]. Many HSP90 clients are known oncoproteins, including EGFR family members, Akt, Bcr-Abl, mutant p53, Kit, and Met, among others[1], [3]. Inhibition of HSP90 function promotes degradation of these client proteins most often through the ubiquitin proteasome pathway ultimately resulting in apoptosis[2], [3], [4]. Selectivity of HSP90 inhibitors for malignant versus normal cells is believed to be conferred by the fact that accumulation of over-expressed and mutated client proteins promotes a shift to the active, super-chaperone complex form of HSP90 in cancer cells[5], [6], [7]. In this state, a client protein associates with HSP90 with the help of co-chaperones such as p23, HSP40, HOP, and HIP[3], [5], [6]. This super-chaperone complex exhibits enhanced ATPase activity, and consequently often binds small molecule ATP mimetics with a higher affinity than the non-complexed form of HSP90, leading to accumulation in tumors relative to normal tissues. As such, the enhanced HSP90 activity confers a greater sensitivity of malignant cells to the loss of HSP90 function[5]. Targeting HSP90 in cancer is also appealing as no resistance mutations have been identified in this protein in human cancers, suggesting it represents a relatively stable target for drug treatment[8]. Because HSP90 inhibition can affect multiple pathways that frequently contribute to the oncogenic process, HSP90 inhibitors have the potential to demonstrate broad activity across multiple tumor types[1], [3].

The first class of HSP90 inhibitors was based on geldanamycin, a benzoquinone ansamycin antibiotic that binds to the N-terminal ATP-binding pocket of HSP90, thereby blocking its ATPase function. Geldanamycin and its semi-synthetic derivatives 17-AAG and 17-DMAG prevent the stabilization of client proteins, ultimately resulting in their degradation[9], [10], [11]. However, geldanamycin and its derivatives have a number of limitations including formulation challenges and side effects such as hepatotoxicity[12]. STA-1474 (Synta Pharmaceuticals Corp, Lexington, MA, USA) is a highly soluble prodrug of ganetespib (formerly STA-9090), a novel resorcinol-containing compound unrelated to geldanamycin that binds in the ATP-biding domain at the N-terminus of HSP90 and acts as a potent HSP90 inhibitor. Ganetespib induces degradation of multiple HSP90 client proteins, killing a wide variety of human cancer cell lines at low nanomolar concentrations *in vitro*, and exhibits potent anti-cancer activity in xenograft tumor models in mice ([13], [14], [15]and unpublished data, Synta Pharmaceuticals Corp.).

In previous studies, we demonstrated that ganetespib and its water soluble prodrug STA-1474 have activity against canine tumor cell lines[14], [15]. Treatment of osteosarcoma (OSA) and mast cell tumor (MCT) cell lines with either ganetespib or STA-1474 induced growth inhibition, apoptosis that was caspase3/7 dependent, and inactivation and/or down-regulation of pKit/Kit, pMet/Met, pAkt/Akt, and pSTAT3. Both ganetespib and STA-1474 exhibited superior activity to the geldanamycin derivative 17-AAG. Ganetespib inhibited tumor growth in a murine MCT xenograft model, while STA-1474 induced tumor regression, caspase-3 activation and downregulation of phospho-Met (pMet)/Met and p-Akt/Akt in OSA xenografts[14], [15]. The purpose of the following clinical trial was to extend our *in vitro* and murine studies and investigate the safety and efficacy of STA-1474 in dogs with spontaneous tumors as a prelude to future clinical work in humans with cancer.

## Materials and Methods

### Eligibility

This clinical trial was approved by the Ohio State University Veterinary Medical Center Hospital Executive Committee in July 2007. Written informed consent from the owner of each dog was requested according to IACUC and The Ohio State University College of Veterinary Medicine guidelines. STA-1474 was administered to dogs with spontaneous tumors that had failed conventional therapy or for which there were no therapeutic alternatives, or for which conventional therapy was not desired by the owner. To be eligible for the study, each dog must have been diagnosed with one of a selected set of histologically confirmed spontaneous malignancies, and have met all of the inclusion criteria and none of the exclusion criteria. Eligible malignancies included carcinomas, sarcomas, mast cell tumors, histiocytic sarcomas, or lymphomas. Additional eligibility criteria included: >1 year old at study entry; adequate organ function; at least 2 weeks since prior chemotherapy or radiation with complete recovery from the acute toxicities of these treatments (3 weeks for a surgical procedure); at least 1 week since prior treatment with any other investigational drug; no diagnosis of leukemia; and no evidence of brain metastases or any serious systemic disorder incompatible with the study at the discretion of the investigator.

### STA-1474 formulation

**Figure 1:**
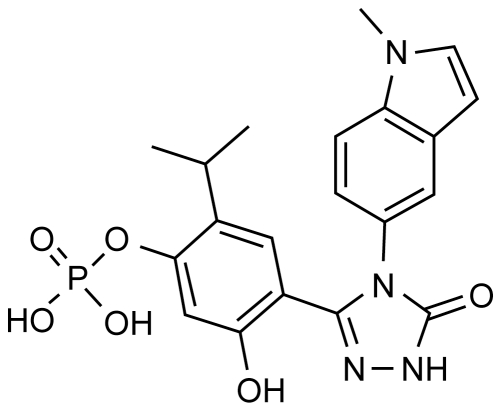
Structure of STA-1474. Shown is the chemical structure of STA-1474, a water-soluble phosphate prodrug of ganetespib.

STA-1474, a soluble prodrug of ganetespib, was provided by Synta Pharmaceuticals Corp (Figure 1). Lyophilized STA-1474 drug product was stored at 4°C and protected from light, until the day of treatment. Drug was dissolved in Plasmalyte 148 USP and pH was adjusted to the range of 6.0 to 7.0, using 0.1 N NaOH standard solution for a final concentration of 3 mg/mL. The drug solution was sterile filtered with 0.2 µ nylon filter and loaded into a non-DEHP fluid path IntraVia^TM^ 250 mL empty container. The loaded infusion bag was then connected to a polyethylene lined vented paclitaxel set, containing a 0.22 µ filter, for administration. Both the infusion bag and line were covered to protect from light. Drug solution was used within 24 hours of preparation.

### Study design

This study was a Phase I dose escalating, open label assessment of the safety and pharmacokinetics of STA-1474 in client owned dogs with spontaneous malignancies. Dogs were administered STA-1474 on one of three dosing regimens and were evaluated weekly or twice per week dependent on the regimen used. Assessment of clinical toxicities and tumor response was performed at each visit. Dogs were evaluated for hematologic and biochemical toxicities every 7 days with routine bloodwork. The initial dose of STA-1474 was set at 7 mg/kg based on previous data from normal laboratory dogs (data not shown) and dose escalation was set at 2.5 mg/kg increments in cohorts of 3 until dose limiting toxicity (DLT) was identified. The DLT was considered to be any grade 3 or 4 hematologic or non-hematologic toxicity based on the established VCOG-CTAE criteria[16]. Additionally, any chronic non-grade 3 or 4 toxicities considered to significantly impair quality of life (i.e., lethargy, inappetence) were qualified as DLTs. Disease progression or signs and symptoms definitely related to disease were not considered adverse events (AEs).

### Toxicity assessment

Each patient underwent a baseline complete history, physical examination, and predose laboratory assessment that included a complete blood count (CBC), serum biochemistry profile, and urinalysis. Patients were assessed for adverse events on days 8, 15, 22, and 29, at which time a complete blood count with differential and clinical chemistry were performed. Urinalysis was performed at baseline and repeated only if indicated. Stipulations regarding minimal hematological requirements to continue dosing were included in the protocol: hematocrit >25%, neutrophils >1500/L, platelets >100,000/L. In addition, liver transaminases were required to be <4X upper limit of normal with a normal total bilirubin and serum creatinine to continue STA-1474 therapy.

### Concomitant medications

To prevent or treat drug-related gastrointestinal toxicities, supportive care was administered as needed to dogs enrolled in the daily dosing cohorts. This typically consisted of famotidine, metronidazole, loperamide, metoclopramide, ondansetron, and/or maropitant. Antihistamines were administered to dogs with mast cell tumors, as these tumors are known to release histamine. Other supportive care administered to dogs consisted of prednisone and non-steroidal anti-inflammatory medications to treat tumor-associated inflammation, inappetence, and for pain control.

### Tumor response assessment

Tumor assessments were completed prior to study entry, and days 8, 15, 22, and 29. For dogs that continued beyond the planned 4 doses of STA-1474, response assessments were performed every 3–6 weeks thereafter, or at the time of suspected tumor progression. Responses were assessed by the investigator according to pre-defined protocol criteria. The response in dogs with assessable disease was performed by clinical examination, radiography, ultrasonography, or CT scans. Many lesions were not amenable for quantitative radiographic imaging, but were followed either by serial clinical examination (superficial lesions; palpable lymph nodes) or by ultrasonography (abdominal lymph nodes). Thoracic lesions were assessed by thoracic radiography and nasal lesions by CT scan.

The response in dogs with measurable disease was judged by the investigator on the basis of RECIST criteria[17]. A complete response (CR) was defined as disappearance of all disease on two measurements separated by a minimum period of 3 weeks. A partial response (PR) was defined as greater than 30% reduction in the sum of the longest diameter of the target lesions documented by two assessments separated by at least 3 weeks. An increase of >20% in the size of all measurable tumor areas as measured by the sum of longest diameters of the target lesions taken as reference the smallest sum since initiation of therapy, or the appearance of any new lesion(s) would qualify as progressive disease (PD). A mixed response (MR) was defined as PR in some target lesions with PD in other target lesions. Stable disease (SD) was defined by the absence of criteria for either a response or progression. Dogs who had no evidence of tumor progression and who had not experienced any unacceptable toxicity were eligible for extended treatment cycles. Dose escalation in an individual dog was permitted if the dog was tolerating therapy.

### Ganetespib pharmacokinetics

Plasma samples were collected on day 1 and day 22 of therapy at the specified intervals: before and after dosing at 30, 60, 90, 120, 180, 300 (5 hr), 540 (9 hr), and 1440 (24 hr) minutes relative to the start of dosing. On day 8, day 15, and day 29, plasma was collected before dosing only. Approximately 1 ml of blood was drawn from an indwelling catheter, placed in a sodium heparin vacuum glass tube, and placed on ice until centrifugation. Blood samples were centrifuged at 3300 rpm for 10 minutes, within 30 minutes of collection. Plasma was transferred by pipette into two cryovials and stored at −80°C until analysis.

Plasma concentrations of ganetespib were determined using a qualified liquid chromatography tandem mass spectrometry (LC-MS/MS) method. Samples were analyzed on an HPLC system consisting of an Agilent HP1100 separation module (Santa Clara, CA) coupled to an AB SCIEX API400 MS/MS detector (Foster City, CA). Stable isotope-labeled ganetespib was used as an internal standard. Sample extraction was performed by a liquid-liquid extraction method with 25 µL plasma samples and 800 µL methyl tertiary butyl ether. The organic phase of samples was evaporated to dryness under nitrogen and reconstituted with water/acetonitrile solution for injection. Chromatographic separation was achieved on a reverse-phase analytical column (4 µm particle, 50×2.0 mm i.d.) with gradient elution of water/0.1% formic acid and acetonitrile/0.1% formic acid at a flow rate of 500 µL/min. The total run time was 4 min. Quantitation was achieved by MS/MS detection in positive ion mode by multiple reaction monitoring. The lower limit of quantitation (LLOQ) was 1.00 ng/mL, with a concentration range from 1.00 to 1000 ng/mL.

The pharmacokinetic parameters for ganetespib were determined from the plasma concentration data using the Noncompartmental Analysis module in WinNonlin, version 5.2 (Pharsight Corporation, St.Louis, MO). The predose samples that measured below the LLOQ (BQL) were treated as zero for the calculations. Maximum concentration (Cmax) and time of maximum concentration (Tmax) were directly determined from the observed concentration-time profiles. The area under the plasma concentration-time curve (AUC) was estimated by the linear trapezoidal rule. The apparent half-life (t_1/2_) was calculated as t_1/2_ = 0.693/λ where λ was the elimination rate constant estimated from the regression of the terminal slope of the concentration versus time curve.

### Evaluation of HSP70 expression

Small biopsies were collected from a subset of patient tumors that were accessible at 0, 7, and 24 hrs post treatment. Biopsies were obtained using a 3 mm punch biopsy instrument or 14 gauge Tru-cut needle. All samples were placed in cryovials, immediately flash frozen in liquid nitrogen, and stored at −80°C until HSP70 analysis. To assess relative HSP70 levels in the collected tumor samples, a commercially available HSP70-enzyme linked immunosorbent assay (ELISA; Assay Designs, Ann Arbor, MI) was performed. Frozen tumor samples were pulverized using a frozen mortar and pestle. The resulting powder was resuspended in liquid nitrogen, collected, then split between two 15 mL conicals; one conical for ELISA and one for Western blot (see method below). Once the liquid nitrogen evaporated away, the samples were allowed to thaw on ice. Samples for ELISA were resuspended in (1X) Extraction Reagent containing 1 µg/mL aprotinin, 1 µg/mL leupeptin, 1 µg/mL pepstatin A, 1 mM PMSF, 1 mM sodium orthovanadate, and10 mM sodium fluoride, using 1 mL for each 0.5 cm^3^ of tissue. Samples were incubated on a rocker for 1 hour at 4°C, centrifuged for 15 minutes at 4°C, and then supernatants were collected. Bradford protein quantification assay was performed on the extracts using BioRad Reagent (BioRad, Hercules, CA). Samples were equalized to 250 ug/mL with (1X) Extraction Reagent, aliquoted, and stored at −80°C until analysis. At a later date, frozen samples where allowed to thaw on ice, then serially diluted with Sample Diluent 2 (provided in kit) to 1∶4, 1∶16, and 1∶32. 100 µL of each dilution was added to the appropriate wells, in duplicate, of the 96-well pre-coated ELISA plate. The ELISA was performed following the manufacturer's instructions, and absorbance was measured within 30 minutes using a Spectramax microplate reader (Molecular Devices, Sunnyvale, CA) with wavelength set at 450 nm and a correction wavelength at 540 nm.

To assess HSP70 levels in peripheral blood mononuclear cells, 8 mL of whole blood was collected before dosing and 24 hours after treatment (in some cases also at 7 hours post treatment) on day 1 and day 22 from the jugular vein into two 4 mL BD Vacutainer CPT tubes (BD, Franklin Lakes, NJ). Samples were kept at room temperature until centrifugation for 1 hour at 1600 RCF, within 2 hours of collection. After centrifugation, the mononuclear cell layer in plasma was transferred to a 15 mL conical. PBMCs were washed by adding 15 mL PBS and centrifuging for 15 minutes at 300 RCF. After two washes, cell pellets were resuspended in 1 mL PBS, and transferred to a 1.5 mL microcentrifuge tube. Cells were pelleted again by centrifuging at 13,000 RCF for 1 minute. Supernatant was removed and cell pellets where flash frozen in liquid nitrogen and stored at −80°C. Immediately prior to analysis, PBMC pellets were allowed to thaw on ice, then each sample was resuspended in 500 µL (1X) Extraction buffer with protease inhibitors. Using the method described above, the samples were analyzed for relative concentrations of HSP70 using the HSP70-enzyme linked immunosorbent assay (Assay Designs, Ann Arbor, MI).

### Evaluation of tumor biopsies for HSP90 expression

Frozen tumor samples were processed as described above, then resuspended in 100–150 uL of lysis buffer consisting of 20 mM Tris-HCl pH 8.0, 137 mM NaCl, 10% glycerol, 1% IGEPAL CA-630, 10 mM ethylenediaminetetraacetic acid (EDTA), 1 mg/mL aprotinin, 1 mg/mL leupeptin, 1 mg/mL pepstatin A, 1 mM phenylmethysulphonyl fluoride, 1 mM sodium orthovanadate, 10 mM sodium fluoride (all from Sigma, St. Louis, MO). Sample were rocked at 4°C for 1 hour, and then centrifuged for 15 minutes at 13,000 RCF. A Bradford protein assay was performed on the lysates using BioRad Reagent (Hercules, CA). 60–100 µg of protein were separated by SDS-page, and transferred to PVDF membranes. The membranes were incubated overnight with anti-HSP90 antibodies (Cell Signaling Technology, Danvers, MA), washed, then incubated with horseradish peroxidase linked secondary antibody. Membranes were washed again, then exposed to Super Signal West Dura Extended Duration Substrate (Pierce, Rockford, IL) Membranes were stripped, washed, and reprobed for β-Actin (Santa Cruz Biotechnology, Santa Cruz, CA) using the SNAP i.d. apparatus (Millipore, Billerica, MA) according to manufacture's instructions.

## Results

### Subjects

A total of 25 dogs with a variety of spontaneous cancers were enrolled in the study. Patient demographics and tumor type are listed in Table 1. The majority of dogs enrolled in this study had failed prior treatment regimens (n = 18, 72%) including surgery, radiation therapy, chemotherapy, or a combination of these treatments. Given the previous data demonstrating biologic activity of ganetespib in canine osteosarcoma (OSA) and mast cell tumor (MCT) cell lines, as well as evidence of tumor regression/stabilization in murine xenograft models of canine OSA and MCT, dogs with these tumor types were enriched in this Phase I study. Tumor types represented included OSA (n = 10), MCT (n = 4), thyroid carcinoma (n = 3), lymphoma (n = 3), other carcinoma (n = 2), nasal chondrosarcoma (n = 1), oral malignant melanoma (n = 1), and oral fibrosarcoma (n = 1).

**Table 1:** Subject demographics.

Characteristics	Total	1 hr 1x/wk	8 hr 1x/wk	1 hr 2x/wk
**Number of dogs**	25	12	6	7
**Age**				
Median	9	9	7.5	9
Range	2–14	5–14	4–9	2–11
**Gender**				
Male intact	1	1		
Male neutered	9	2	3	4
Female neutered	15	9	3	3
**Tumor Type**				
Osteosarcoma	10	5	3	2
Mast cell tumor	4	1	1	2
Thyroid carcinoma	3	1	2	
Lymphoma	3			3
Other carcinoma	2	2		
Nasal chondrosarcoma	1	1		
Oral melanoma	1	1		
Oral fibrosarcoma	1	1		
**Prior Treatment**				
Yes	18	8	6	4
No	7	4		3

### Treatment groups and pharmacokinetic analysis

Dogs (n = 12) were initially entered into a treatment regimen consisting of STA-1474 administered intravenously over one hour once per week. A second cohort of dogs (n = 6) was administered STA-1474 over 8 hours once per week. This dosing regimen was chosen based on a partial response to therapy observed in one dog with malignant melanoma in the first cohort following drug extravasation and prolonged drug exposure due to slow subcutaneous absorption. The third cohort of dogs (n = 7) received STA-1474 administered over one hour twice per week. Each dog entered into the study was intended to receive at least 4 doses of STA-1474 with the option to continue treatment in the face of either stable disease or an objective tumor response.

**Figure 2:**
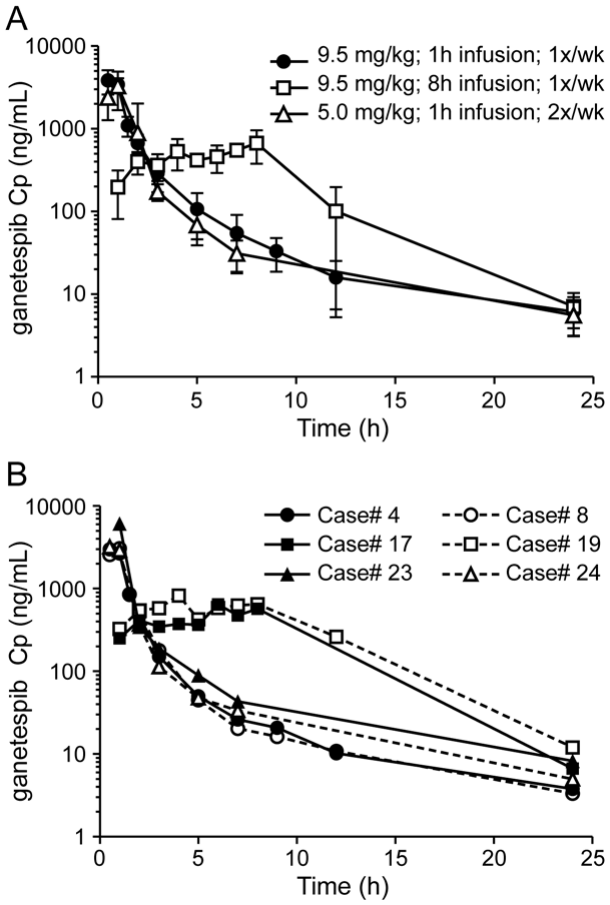
Pharmacokinetic analysis of STA-1474 following administration using three different dosing regimens. a) Mean ganetespib plasma concentration – time profile for each dosing regimen b) Representative ganetespib plasma concentration – time profiles for two patients in each dosing regimen.

Pharmacokinetic data are summarized in Table 2. Full pharmacokinetic sampling was performed for all dogs in this study on the first day of drug administration. The mean plasma concentration – time profiles for dogs in the three treatment groups are shown in Figure 2a, with representative profiles for 6 dogs (2 in each treatment group) in Figure 2b. For dogs receiving any of the three dosing regimens, the AUC was relatively similar, ranging between 5013–5701 ng/ml-hr ganetespib. However, the hourly drug infusions at 9.25 mg/kg or 5 mg/kg resulted in a shorter Tmax (0.7 hr and 1.1 hr, respectively) and higher Cmax (3982 ng/ml and 3415 ng/ml, respectively) compared to the 8 hour infusion (Tmax 5.7 hr, Cmax 802 ng/ml).

**Table 2:** Mean pharmacokinetic parameters of STA-9090.

STA-1474 Dose	Infusion	Treatment	t_1/2_	Tmax	Cmax	AUC_last_
(mg/kg)	(h)	Frequency	(h)	(h)	(ng/mL)	(ng/mL·h)
9.5	1	Once weekly	5.6	0.7	3982	5701
9.5	8	Once weekly	NR*	5.7	802	5605
5	1	Twice weekly	6.1	1.1	3415	5013

*NR: not reportable due to insufficient terminal phase characterization.

### Safety evaluation

Adverse events during treatment cycles (summarized in Table 3) consisted nearly entirely of gastrointestinal upset (anorexia, nausea, vomiting, diarrhea) and lethargy. No drug-related hematologic or biochemical toxicities were noted at any doses. One dog with metastatic osteosarcoma developed acute anuric renal failure resulting in euthanasia. Subsequent necropsy revealed the presence of large tumor emboli in both renal arteries, with no evidence of drug induced changes to the renal parenchyma, and as such progressive disease was determined to be the cause of the observed renal failure. A second dog developed acute liver function test (ALT, ALP) and bilirubin elevations 24 hours following drug administration. The dog subsequently vomited a large foreign body consisting of gauze pads used for bandaging of the catheter site and the biochemical values returned to normal within 72 hours; a partial temporary obstruction of the common bile duct due to foreign body was determined to be the cause of the biochemical changes in this case.

**Table 3:** Enrollment and toxicity by dose/regimen.

Regimen	Dose (mg/kg)	No. of dogs	Lethargy			Anorexia			Vomiting			Diarrhea		
			1	2	3	1	2	3	1	2	3	1	2	3
1 hr/week	7.0	4	2				2		3			2	3	
	9.5	5		1	1					2		1		1
	10.25	3		3			3			1			3	
8 hr/week	9.5	6	2			5	4			3		4	4	
1 hr/2x week	5.0	7	4	2		3	5					5	1	1

Dose escalation was performed and dose limiting toxicities were identified using the once per week hourly infusion. While nearly all gastrointestinal toxicities were primarily grade 1 or 2 in nature, at doses above 9.5 mg/kg, dogs were found to have lethargy and anorexia that owners felt significantly impaired quality of life, resulting in discontinuation of drug administration. For example, all three dogs that received 10.25 mg/kg of drug experienced grade 2 diarrhea, lethargy, and anorexia and each subsequently received only one dose of drug. As such, 9.5 mg/kg was considered to be the maximum tolerated weekly dose of drug when administered weekly over 1 or 8 hours. At this dose, the gastrointestinal toxicities were manageable with the addition of concomitant medications consisting of ondansetron, metoclopramide and/or maropitant for nausea/vomiting and metronidazole and/or loperamide for diarrhea. All dogs that continued on therapy with STA-1474 beyond the initial 4 cycles of treatment remained on concomitant medications for the duration of drug treatment.

### Response to therapy

**Figure 3:**
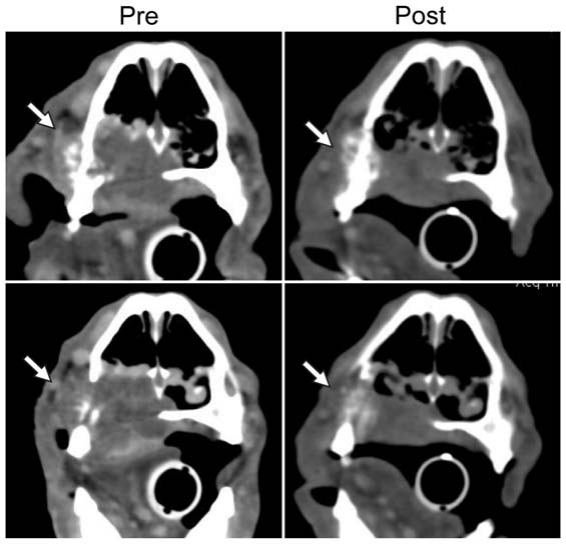
Serial CT demonstrating regression of oral malignant melanoma following treatment with STA-1474. Patient # 5 had an aggressive oral malignant melanoma that had invaded into the nasal cavity. During the 4^th^ treatment cycle with STA-1474, an extravasation event occurred resulting in altered drug pharmacokinetics. A marked decrease in the oral mass was observed 7 days later and a subsequent CT scan confirmed a partial response to therapy. Shown are two representative matched CT images of tumor before and after treatment.

Response rates were determined for dogs entered into the 3 different dosing groups. For dogs that received once per week dosing regimen (n = 12), no objective responses were noted with the exception of one dog with an extensive oral malignant melanoma that experienced an extravasation event during the 4^th^ treatment with STA-1474. This dog had a partial response in tumor size and following pharmacokinetic analysis (Figure 3), it was determined that the inadvertent subcutaneous administration resulted in an extended Tmax, without a significant change in the actual AUC (data not shown).

**Figure 4:**
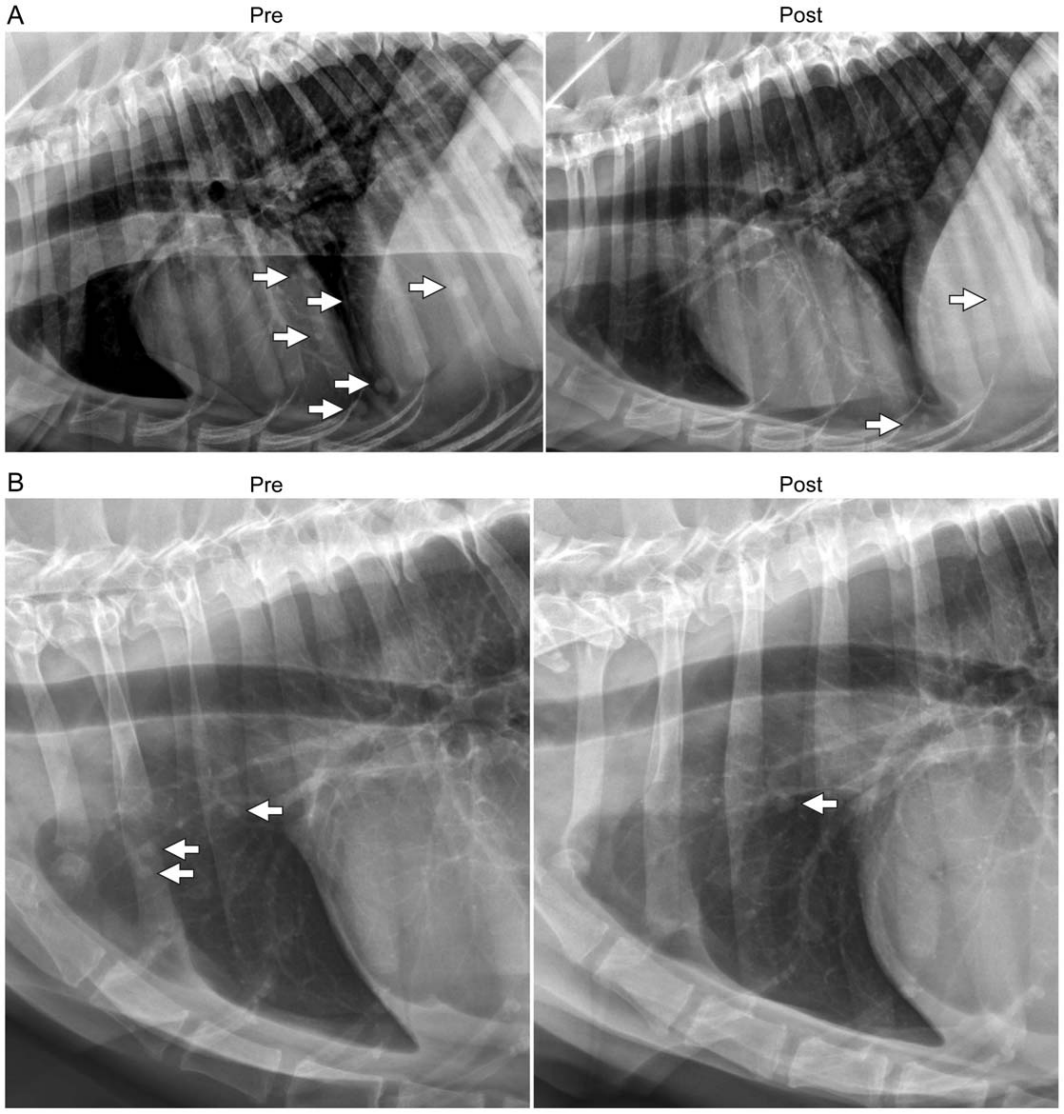
Response of metastatic thyroid carcinoma and OSA to STA-1474 treatment. a) Patient #14 had a locally recurrent thyroid carcinoma with metastatic disease to the lungs. This patient received STA-1474 on the 8 hour infusion protocol and experienced a partial response to therapy of both the locally recurrent and metastatic disease. Shown are representative radiographs before and after 15 treatments with STA-1474. The yellow arrows point to the metastatic pulmonary nodules. b) Patient #18 had metastatic OSA to the lungs following amputation and chemotherapy for an appendicular OSA. This patient received STA-1474 on the 8 hour infusion protocol and experienced a partial response to therapy. Shown are representative radiographs before and after 4 treatments with STA-1474. The yellow arrows point to the metastatic pulmonary nodules.

Based on evidence of biologic activity associated with a longer Tmax, pharmacokinetic modeling was employed to determine a dosing model that would achieve a similar Tmax when drug was delivered over a longer time period at the same dose rate. It was predicted that 9.5 mg/kg of STA-1474 given over an 8 hour infusion would result in plasma concentrations of ganetespib above 100 ng/ml of plasma for 8–10 hours and consequently the next 6 dogs were entered into a cohort evaluating this dosing regimen. Additionally, two dogs from the first dosing cohort (9.5 mg/kg once per week) were switched to the 8 hour infusion protocol after finishing the planned 4 doses of drug. Of the 6 dogs initially treated with the longer infusion, there were two partial responses (metastatic thyroid carcinoma, 36 weeks, Figure 4a; metastatic OSA, 20 weeks, Figure 4b), one mixed response (metastatic MCT, 16 weeks), and two dogs experienced stable disease (both with metastatic OSA, 12 weeks). One dog with metastatic thyroid carcinoma that switched from the 1 hr to 8 hr infusion experienced stable disease for 40 weeks, and the dog with oral malignant melanoma and extravasation event experienced a continued partial response for 12 weeks while on the 8 hour infusion protocol.

**Figure 5:**
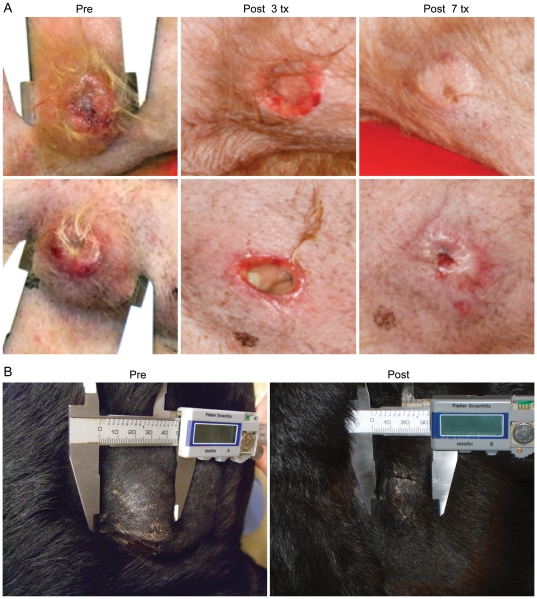
Response of cutaneous mast cell tumors to STA-1474 treatment. a) Patient #19 had recurrent grade 3 cutaneous MCTs. This patient received STA-1474 on the 1 hour infusion protocol twice per week and experienced a partial response to therapy of the cutaneous lesions after 7 treatments. b) Patient #24 had a large previously untreated cutaneous MCT. This patient received STA-1474 on the 1 hour infusion protocol twice per week and experienced a partial response to therapy of the cutaneous lesions after 4 treatments.

Since the longer infusion protocol appeared to be associated with biologic activity, we wanted to determine whether more frequent administration of STA-1474 using the 1 hour infusion protocol would be both tolerable and induce objective responses to therapy. A dose of 5 mg/kg administered over one hour twice per week was chosen based on preliminary data of tolerability in normal dogs and pharmacokinetic modeling (data not shown). As shown in Figure 2a and b, this dosing regimen resulted in a Cmax and AUC slightly lower than that generated by the 9.5 mg/kg dose. Toxicities observed were similar to those with the once weekly higher dose of STA-1474. Of the 7 dogs administered this protocol, 2 experienced partial responses to therapy (both MCTs, Figure 5) and both dogs went on to have their remaining disease surgically excised. One dog (metastatic OSA) experienced stable disease for a short period of time but the owner elected not to continue therapy and as such, this did not meet RECIST criteria. The remaining dogs (lymphoma, n = 3; metastatic OSA, n = 1) experienced progressive disease.

### Evaluation of HSP90 and HSP70 expression in tumor samples and PBMCs

**Figure 6:**
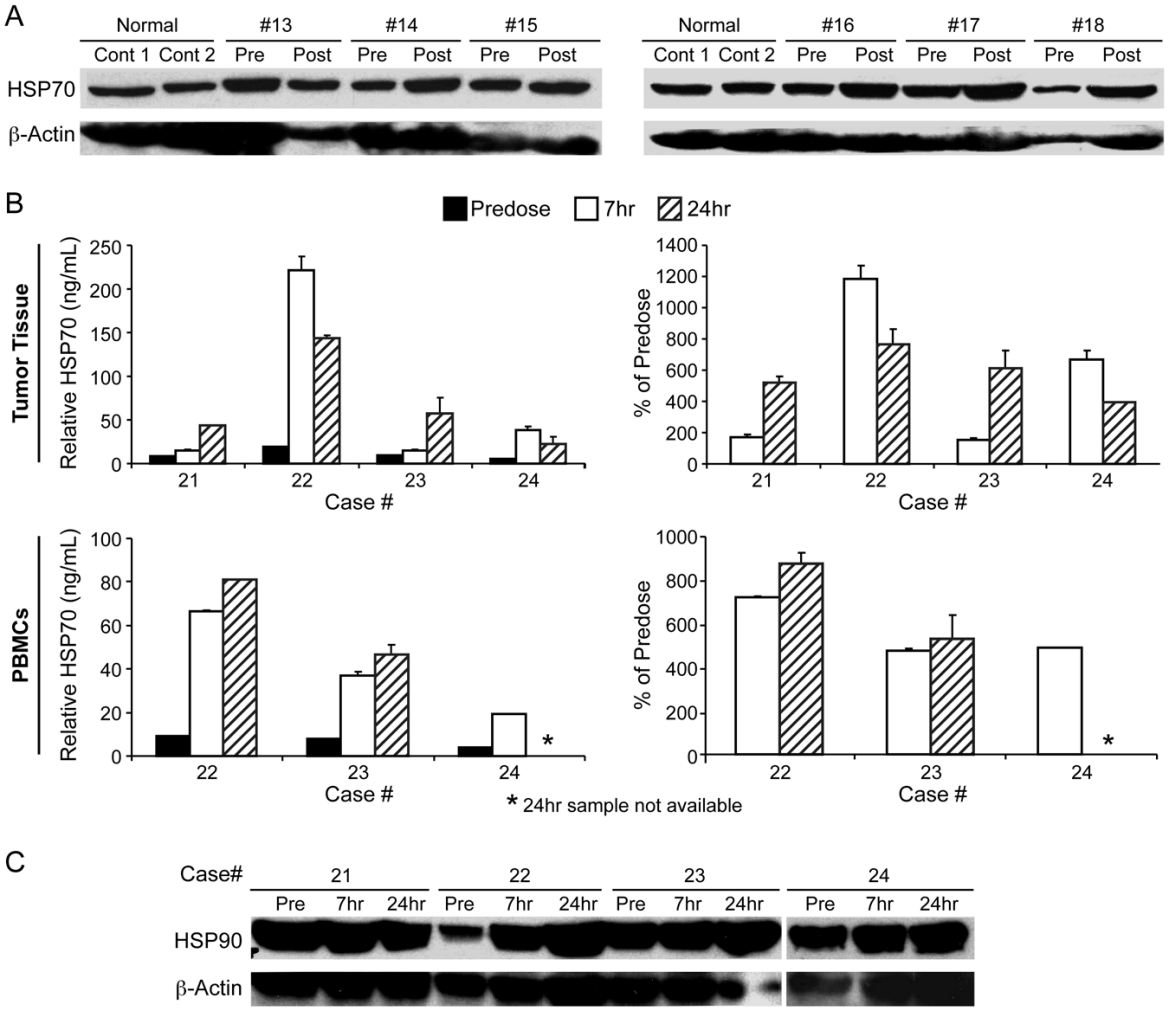
Analysis of HSP90 and HSP70 expression in dogs before and after treatment with STA-1474. a) PBMCs were collected from normal control dogs (cont 1 and cont 2) and study dogs before and 24 hours after treatment with STA-1474 at 9.5 mg/kg over 8 hours. Protein lysates were generated and following SDS-PAGE, Western blotting was performed for HSP70. Blots were then stripped and reprobed for β-actin. b) Tumor biopsies and PBMCs were collected from dogs treated with 5 mg/kg STA-1474 over 1 hour before treatment and at 7 and 24 hours post treatment. Protein lysates were generated and an ELISA was performed to detect HSP70. Results are expressed as HSP70 protein (ng/ml) and percent of baseline. c) Protein lysates generated from the tumor biopsies described above were subjected to SDS-PAGE followed by Western blotting for HSP90. Blots were then stripped and re-probed for β-actin.

Following inhibition of HSP90, rapid upregulation of HSP70 expression is often noted[14], [15], [18]. As such, HSP70 upregulation can potentially serve as a biomarker of loss of HSP90 function. To determine whether treatment with STA-1474 altered the expression of HSP70, and if HSP70 could serve as a potential sensitive surrogate biomarker for HSP90 inhibition PBMCs were collected pre and post treatment and Western blotting for HSP70 was performed. As shown in Figure 6a, small increases in HSP70 expression were apparent in the post treatment PBMC samples, although these changes were difficult to accurately quantitate. Consequently, an ELISA assay was used on subsequent samples to more definitively assess upregulation of HSP70. Figure 6b demonstrates that upregulation of HSP70 in the tumors and PBMCs was more accurately assessed using the ELISA assay. Moreover, these data indicate that changes in HSP70 expression within PBMCs following STA-1474 treatment can be used as a surrogate marker of HSP70 upregulation within tumors. Lastly, HSP90 was expresed in all tumor samples and expression levels did not appear to change substantially 24 hrs following treatment with STA-1474 (Figure 6c).

## Discussion

HSP90, a molecular chaperone with several client proteins known to contribute to tumorigenesis, has arisen as a promising target for therapeutic intervention in a variety of cancers. Biologic activity of the benzoquinone ansamycin antibiotic geldanamycin and its semi-synthetic derivatives 17-AAG and 17-DMAG has been demonstrated *in vivo* and in murine xenograft models for various hematopoietic neoplasms and solid carcinomas[19], [20], [21], [22]. Additionally, several clinical trials evaluating geldanamycin derivatives and other novel HSP90 inhibitors have been completed and several are ongoing. However, despite encouraging results *in vitro* and in mouse models, biologic activity of many of the HSP90 inhibitors has not been as significant as predicted based on experimental models. The reasons for this are multi-factorial and include the fact that inhibition of HSP90 results in upregulation of other chaperones or co-chaperones (HSP70/HSP27) capable of influencing protein stabilization[23]; co-chaperones CDC37, AHA1 and p23 are highly expressed in cancer cells and may enhance the function of HSP90 in the presence of inhibitors[24], [25], [26]; mitochondrial HSP90 is usually not affected by the HSP90 inhibitors now in clinical trials[27], [28]; current dosing/administration schedules may not provide sufficient inhibition of tumor HSP90 activity[1]; and tumor cell reliance on driver proteins (HER2, Kit, etc) that are HSP90 clients may be required for substantial anti-tumor activity. Given that significant barriers to the effective use of HSP90 inhibitors in the clinical setting still exist, a spontaneous tumor model that more closely recapitulates human cancers would be useful to begin to explore treatment strategies more likely to result in clinical benefit. As such, the purpose of this clinical study was to evaluate the safety and activity of STA-1474, a water soluble prodrug of the resorcinol-containing compound ganetespib, in pet dogs with spontaneous cancers.

Ganetespib possesses a number of advantages over geldanamycin and its semi-synthetic derivatives 17-AAG and 17-DMAG including lack of hepatotoxicity and activity at lower drug concentrations. In our previous work with ganetespib and its prodrug STA-1474, we demonstrated that HSP90 inhibition resulted in downregulation of client proteins Kit, Met, and Akt in canine MCT and OSA cell lines, resulting in apoptosis[14], [15]. Biologic effects of ganetespib were demonstrated at concentrations at least 1 log lower than those for 17-AAG. Furthermore, we were able to demonstrate anti-tumor activity of ganetespib against canine MCT and OSA xenografts including downregulation of Met and Akt *in vivo*. STA-1474 was chosen for further study in pet dogs with cancer due to its solubility in water which facilitated administration in the clinical setting.

The demographics of subjects in the study population were similar to those for dogs enrolled in previously reported Phase I clinical trials of novel agents[29]. However, there was an emphasis placed on enrolling dogs with either OSA or MCT given the prior studies *in vitro* and mouse models suggesting HSP90 inhibition would likely have biologic activity in these tumor types. Nearly all adverse events observed in this study were gastrointestinal in nature (anorexia, lethargy, vomiting, and diarrhea) and most were readily manageable with concomitant medications. Importantly, there was no evidence of hematologic toxicity at any of the doses or regimens used indicating that it may be possible to combine ganetespib with other anti-tumor agents that typically exhibit myelosuppression (i.e., chemotherapeutics) without enhanced toxicity. Seven of the 25 dogs (28%) received more than 8 doses of STA-1474 (range 10–38) with the longest dog on study receiving 38 treatments. No hematologic or biochemical consequences of long-term administration of STA-1474 were observed in these dogs, again indicating that prolonged HSP90 inhibition with ganetespib did not affect the bone marrow stem cell compartment or result in liver or renal toxicity.

While objective responses to therapy were observed in this study, nearly all occurred using a dosing regimen that was not originally planned. A dog with oral melanoma in the first dosing cohort received most of the drug subcutaneously as the intravenous catheter was dislodged during the course of administration. Pharmacokinetic analysis revealed an extremely long Tmax associated with sustained blood levels of STA-9090 between 200–600 ng/ml for 8–10 hours. As this was the only patient within the first dosing cohort that exhibited a biologic response to therapy, an additional treatment group was added using an infusion protocol designed to replicate the extravasation event. The objective response rate in this second group was 30% (2PR/6) with a biologic activity rate of 83% (2PR, 1 MR, 2 SD). Importantly, 3 dogs with metastatic OSA derived clinical benefit from STA-1474 treatment in the 8 hour dosing group as opposed to the 7 dogs with OSA that did not derive benefit when treated with the 1 hour dosing regimens. It is therefore possible that longer infusion of STA-1474 induced more sustained inhibition of HSP90 function resulting in enhanced biologic activity.

We documented rapid upregulation of HSP70 expression in both tumor samples and PBMCs that occurred within 7 hours following STA-1474 treatment. It is known that HSF-1-dependent HSP70 and HSP27 upregulation frequently occurs following loss of HSP90 function[23], [30], [31]. HSP70 inhibits multiple aspects of the apoptotic cascade, including release of cytochrome C and apoptosis-inducing factor (AIF), nuclear import of AIF, and activation of procaspases 9 and 3 and as such, its upregulation may also act to protect tumor cells from apoptosis[32]. Recent evidence indicates that upregulation of HSP70 and HSP27 may promote resistance to HSP90 inhibitors as silencing of HSP70 and/or HSP27 significantly increases tumor cell sensitivity to geldanamycin[30], [31]. The effects of combined HSP90/HSP70 inhibition have not yet been investigated in the clinical setting, but such an approach may provide more durable anti-tumor activity. Indeed, although partial responses to therapy were observed in our study, no complete responses occurred and all dogs that experienced a PR or SD eventually developed progressive disease.

The biologic activity of STA-1474 we observed in dogs with metastatic OSA is important as this disease serves as a relevant model of OSA in humans[33], [34]. In both dogs and humans, no significant improvements in survival have occurred over the past 10–15 years and chemotherapy resistant metastases are common in both species. The 5 year survival rate for humans with OSA treated with surgical resection and adjuvant chemotherapy remains at 60–70% while the 2 year survival rate for dogs with appendicular OSA treated with amputation and standard chemotherapy is only 10–20%[33], [35], [36]. It is possible that the addition of chemotherapy to STA-1474 treatment may promote enhanced cell killing of OSA cells, as loss of HSP90 function may induce rapid modulation of key cell signaling proteins vital for apoptosis protection thereby sensitizing these cells to DNA damaging agents. We are currently investigating the effects of combined or sequential treatment of OSA cell lines with chemotherapy (doxorubicin, platinum agents) and ganetespib to determine if synergistic interactions can be identified. Additionally, recent data suggest that loss of proteasome function may enhance the effects of HSP90 inhibition[37]; potential synergistic interactions of bortezimib and STA-1474 are therefore being explored. Lastly, we did observe an objective response to STA-1474 in a dog with oral melanoma. Activity of HSP90 inhibitors has been observed against metastatic melanoma in human patients, and there is now evidence suggesting a role for phospholipase A2 activation in the Hsp90 inhibitor-induced metabolic response[38]. This deserves further evaluation in melanoma as well as other solid tumors as it may represent another mechanism through which synergistic anti-tumor activity can be achieved.

In summary, we describe a preclinical proof-of-concept Phase I study of STA-1474, a water-soluble prodrug of the potent HSP90 inhibitor ganetespib in a relevant large animal model of spontaneous naturally occurring cancer. This study provided important information regarding expected gastrointestinal adverse events likely to occur in human clinical trials with ganetespib, evidence that hematologic and biochemical toxicities are unlikely to arise during therapy, demonstration of biologic activity in a pretreated patient population, and importantly, tolerability of drug administration over multiple cycles of therapy. These data laid the groundwork for the current clinical studies of ganetespib in humans with cancer.
